# Accuracy of handgrip and respiratory muscle strength in identifying sarcopenia in older, community-dwelling, Brazilian women

**DOI:** 10.1038/s41598-023-28549-5

**Published:** 2023-01-27

**Authors:** Luana Aparecida Soares, Liliana Pereira Lima, Ana Caroline Negreiros Prates, Arthur Nascimento Arrieiro, Leonardo Augusto Da Costa Teixeira, Tamiris Campos Duarte, Jousielle Márcia dos Santos, Vanessa Kelly da Silva Lage, Fabiana Angélica de Paula, Henrique Silveira Costa, Pedro Henrique Scheidt Figueiredo, Vike Maria Tamar Leão de Almeida, Núbia de Sara Abreu, Sabrina Paula Costa, Franciane Pereira Brant, Rávylla Rúbia Lima, Ronaldo Luis Thomasini, Leani Souza Máximo Pereira, Fabiana Souza Máximo Pereira, Adriana Netto Parentoni, Núbia Carelli Pereira de Avelar, Amanda Aparecida Oliveira Leopoldino, Vanessa Amaral Mendonça, Ana Cristina Rodrigues Lacerda

**Affiliations:** 1 Departamento de Fisioterapia, Universidade Federal dos Vales do Jequitinhonha e Mucuri, Diamantina, Minas Gerais Brazil; 2 Programa de Pós-Graduação em Reabilitação e Desempenho Funcional, Universidade Federal dos Vales do Jequitinhonha e Mucuri, Diamantina, Minas Gerais Brazil; 3 Programa de Pós-graduação Multicêntrico em Ciências Fisiológicas, Universidade Federal dos Vales do Jequitinhonha e Mucuri, Diamantina, Minas Gerais Brazil; 4 Programa de Pós-Graduação em Ciências da Saúde, Universidade Federal dos Vales do Jequitinhonha e Mucuri, Diamantina, Minas Gerais Brazil; 5 Faculdade de Medicina, Universidade Federal dos Vales do Jequitinhonha e Mucuri, Diamantina, Minas Gerais Brazil; 6 Universidade Federal de Minas Gerais, Belo Horizonte, Minas Gerais Brazil; 7 Universidade Federal de Santa Catarina, Santa Catarina, Brazil; 8 Faculdade Ciências Médicas de Minas Gerais, Minas Gerais, Brazil

**Keywords:** Health care, Diagnosis, Geriatrics

## Abstract

Certain cut-off points for sarcopenia screening and diagnosis are arbitrary and based on European populations, with normative references often obtained from healthy young adults. Although respiratory skeletal muscle strength tests represent low-cost clinical measures commonly performed in clinical practice by health professionals, a gap remains regarding whether respiratory skeletal muscle strength tests are adequate and sensitive measures for sarcopenia screening. This study aimed to verify the value of handgrip and respiratory muscle strength as possible discriminators to identify sarcopenia and to establish cut-off points for sarcopenia screening in community-dwelling, Brazilian women. In a cross-sectional study, 154 community-dwelling, Brazilian women (65–96 years) were assessed for appendicular skeletal muscle mass, handgrip (HGS), and respiratory muscular strength, including maximal inspiratory pressure (MIP) and maximal expiratory pressure (MEP). The data were analyzed using the ROC curve and the Youden Index determined cut-off points. Statistical significance was set at 5%. 88 participants (57%) were sarcopenic. MEP (OR 0.98 [95%CI 0.97, 1.00], p = 0.023) and HGS (OR 0.82 [95% CI 0.75, 0.90], p < 0.001) were independent factors for sarcopenia in older. The optimal cut-off points for identifying sarcopenia were ≤ 77 cmH_2_O for MEP (AUC = 0.72), and ≤ 20 kg for HGS (AUC = 0.80). Simple muscular strength tests, including HGS and MEP, may be considered in the identification of sarcopenia in older, community-dwelling, Brazilian women. Future work is still needed to assess external validation of the proposed cut-offs before the clinical application.

## Introduction

Sarcopenia is a progressive and generalized skeletal muscle disorder associated with increased likelihood of adverse outcomes, including falls, fractures, functional disability, physical performance and skeletal muscle strength impairment, mortality, and others^1^. In financial terms for healthcare systems, the presence of sarcopenia increases the risk of hospitalization and the subsequent cost of care during hospitalization^2^. In recent decades, there has been a dramatic increase in the prevalence of sarcopenia due to the aging population. In Brazil, sarcopenia prevalence in community-dwelling and hospitalized older people is estimated to be around 13.9–21.8%^3–5^.

There is a wide variety of tests and tools available for sarcopenia stratifications in clinical practice and in research^6,7^. According to the European Working Group on Sarcopenia in Older People (EWGSOP2), sarcopenia diagnosis is based on parameters of skeletal muscle mass and strength, and physical performance^1^. In clinical practice, for sarcopenia screening and diagnosis, EWGSOP2 recommends the use of muscle strength tests such as handgrip strength (HGS) to identify probable presence of sarcopenia^8^. HGS is an easy measure with practical applicability and is associated with lower limb strength and disability^9^. The use of dual-energy X-ray absorptiometry (DXA) is recommended to detect low skeletal muscle quantity^10^. In addition, physical performance measures are often used to determine sarcopenia severity^1^.

As certain cut-off points for sarcopenia screening and diagnosis are arbitrary and based on European populations, with the normative references often being obtained from healthy young adults, studies have recommended caution in adopting cut-off points for populations with different genotypic and phenotypic characteristics^11–13^. Thus, normative data obtained from appropriate reference populations should be used to determine specific cut-off points^14,15^. Therefore, to identify older adults at risk of sarcopenia^15,16^, it is crucial to use appropriate, specific cut-off points for the target population.

HGS measurement has been commonly used to indirectly screen and classify the presence of sarcopenia^8^. However, in the screening and classification of the presence of sarcopenia in women, a wide range of values from 16 to 20 kg have been adopted as cut-off point^8,17–20^. In this sense, there is, no known study evaluating the effectiveness of HGS in identifying low muscle mass for screening and diagnosis of sarcopenia in older, community-dwelling, Brazilian women. Therefore, a gap remains regarding the cut-off point for sarcopenia screening in the aforementioned population.

Other strength tests have also been suggested to discriminate sarcopenia, such as maximal respiratory pressures. As in the case of HGS, they were previously related to peripheral muscle strength^21^ and sarcopenia indicators^22–24^. Thus, maximal inspiratory pressure (MIP) and maximal expiratory pressure (MEP) may represent additional tools to HGS in the screening and diagnosis of sarcopenia.

Although respiratory skeletal muscle strength tests represent low-cost clinical measures commonly performed in clinical practice by health professionals, a gap remains regarding whether respiratory skeletal muscle strength tests are adequate and sensitive measures for sarcopenia screening and diagnosis. Hence, the aim of this research was to evaluate the association of respiratory muscle strength and HGS with the presence of sarcopenia in older, community-dwelling, Brazilian women and to establish cut-off points for sarcopenia screening, thereby facilitating early detection and better management of sarcopenia in clinical practice.

## Methods

### Patients and methods

This was a cross-sectional study involving participants evaluated at the Laboratório de Fisiologia do Exercício (Exercise Physiology Laboratory) (LAFIEX) at the Universidade Federal dos Vales do Jequitinhonha e Mucuri (Federal University of the Jequitinhonha and Mucuri Valleys) (UFVJM), from June 2016 to June 2017. The research was approved by the Institutional Ethics Committee (UFVJM; identification number 1.461.306) and carried out according to the Declaration of Helsinki^25^. All patients gave their written informed consent before participating in the study and were evaluated consecutively. The present study was edited following the guidelines of the STARD (Standards for Reporting of Diagnostic Accuracy Studies) statement^26^, when applicable.

Older, community-dwelling adults were recruited in their homes and invited to participate in the study. Following application of the inclusion and exclusion criteria, the eligible volunteers were subjected to previously scheduled assessments at LAFIEX, including body composition analyses and muscular strength tests (HGS, MIP, and MEP).

The inclusion criteria were older women, aged 65 or over, regardless of race or social class and who were willing to participate in the research. The exclusion criteria were cognitive impairment, detected by the Mini-Mental State Examination, according to schooling; being unable to walk independently without the aid of a walking device; having been hospitalized or having suffered fractures in the last 3 months; using the medication digoxin, due to its positive influence on respiratory strength^27^; inflammatory disease in the acute phase; thyroid dysfunction; neoplasm in activity in the last 5 years; being in palliative care; using anti-inflammatory drugs or drugs that act on the immune system; performing physical activity on a regular basis (at least three times a week); severe visual and auditory impairment; and acute cardiorespiratory diseases.

The sample size for determining a suitable cut-off value was obtained considering 1.96 as the percentage for the normal distribution, area under the curve of 0.8, confidence interval of 0.05, and a ratio of 1 between number of participants with and without sarcopenia^28^. Thus, the sample size was estimated at 154 participants. In addition, at least 77 participants should be sarcopenic in the estimated sample.

### Measurements

#### Body composition and diagnosis of sarcopenia

Body mass and height were measured using scales with a stadiometer. Body mass index (BMI) was calculated by body weight (kg) divided by height squared (m^2^). Assessment of lean mass, fat mass, and bone mineral density, among others, was performed using dual-energy X-ray absorptiometry—DXA (Lunar Radiation Corporation, Madison, Wisconsin, USA, model DPX), which is considered a suitable and ideal measure in the research setting, clinical practice, and primary health care for confirming sarcopenia diagnosis^9^. Thus, DXA was the reference standard. The diagnosis of sarcopenia was made considering the cut-off points of Appendicular Skeletal Muscle Mass (ASM), measured as the sum of the non-bone and non-fat mass of the four limbs^10,19^. The reference value of 15 kg, relative to ASM, was used as a cut-off point for the detection of sarcopenia and participants were classified as non-sarcopenic and sarcopenic^1,29^.

#### Handgrip strength

Handgrip strength was used as the index test and evaluated using the Jamar^®^ dynamometer, measured in kg, by means of an isometric contraction applied over its loops, according to the American Society of Hand Therapists (2002)^30^. Three measurements were performed with the dominant hand and their average was used for analysis. An interval of one minute was given between each measurement^31^.

Respiratory muscle strength.

Respiratory muscle strength was assessed by measuring the maximal inspiratory pressure (MIP) and the maximal expiratory pressure (MEP) using a hand vacuum pump, model MV-150/300, manufactured by Ger-Ar Comércio e Equipamentos Ltda^®^. Each volunteer was seated with their feet supported and their nose occluded with a nasal clip. The maneuvers were repeated up to five times. Three acceptable maneuvers were collected and the maximal respiratory efforts sustained for at least 2 s. The measurements considered acceptable were those without air leaks and with a variation ≤ 10% from the highest value detected^32^. The sequence of MIP and MEP measurements was random, and the highest measurement was selected for analysis. The MIP was measured based on the residual volume, and the MEP on total lung capacity^33^. An interval of at least 1 min was established between each MIP and MEP measurement to allow the participant to recover. For precision in the interval between MIP and MEP collection, normalization of O_2_ saturation and the return of systemic blood pressure to basal levels were observed^34^.

### Statistical analysis

Statistical analyses were performed using the Statistical Package for the Social Sciences version 22.0 (SPSS Statistics; IBM, Armonk, NY) and MedCalc Statistical Software version 13.1 (MedCalc Software, Ostend, Belgium). The data distribution was verified using the Kolmogorov–Smirnov test. Continuous variables were shown as mean and standard deviation (normal distribution) or median and interquartile range (non-normal distribution). Comparisons between groups were performed through independent t-test and Mann–Whitney’s test. Univariate and multivariate logistic regression analyses were used to examine the association between muscular strength tests (MIP, MEP, HGS) and sarcopenia. The receiver operating characteristic (ROC) curves were used to test the sensitivity and specificity of MIP, MEP, and HGS tests in identifying sarcopenia. The area under the ROC curve (AUC) and 95% confidence interval (CI) were calculated for all tests and optimal cut-offs were determined using the Youden Index. An AUC greater than 0.7 was considered acceptable, while an area greater than 0.8 was considered excellent for the propose the cut-offs points^35^. Statistical significance was set at 5%.

### Ethics approval and consent to participate

Ethics approval has been granted by the Institutional Ethics Committee (Universidade Federal dos Vales do Jequitinhonha e Mucuri, Diamantina, Brazil; identification number 1.461.306). Informed consent was obtained from all study participants.

## Results

### Characteristics of subjects

**Figure 1:**
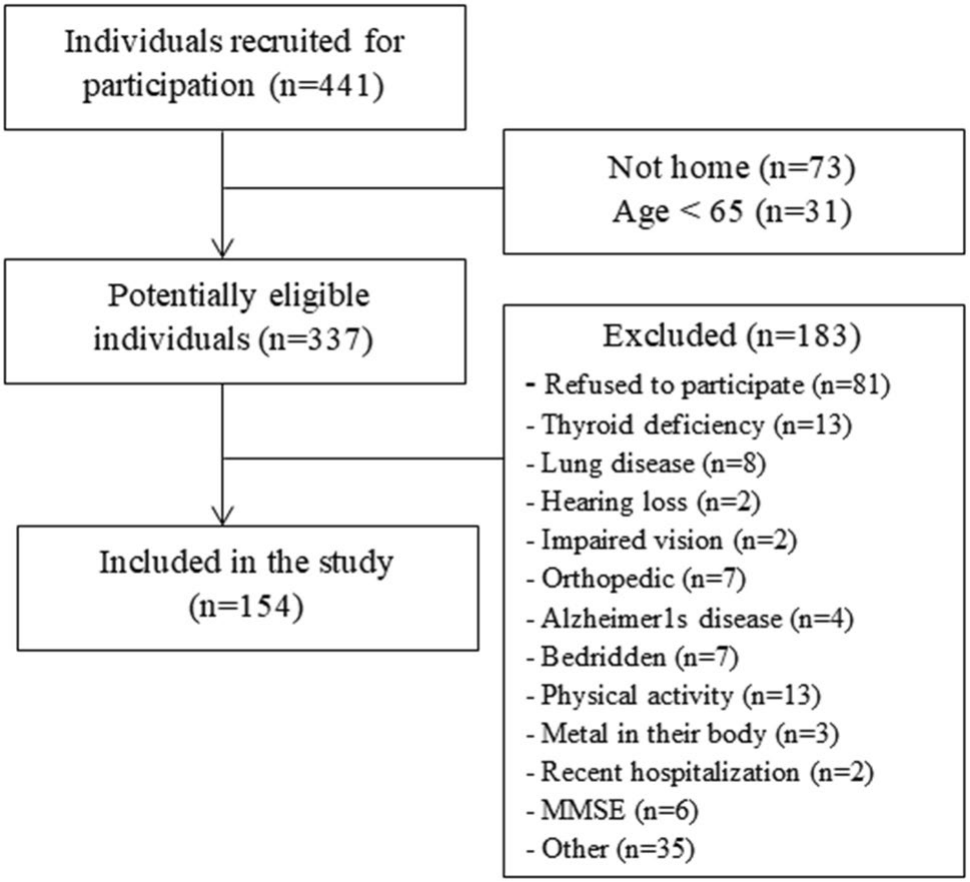
Flowchart of the study.

Four hundred and forty-one patients were initially recruited for participation. After applying the exclusion criteria, a 154 elderly community residents were enrolled in the study (Fig. 1).

Characteristics of the participants are presented in Table 1. There were differences in age, as well anthropometric and body composition variables between the groups. Thus, the sarcopenic group were older, with lower BMI, lean mas, fat mass, and BMD. There was also a statistical difference in strength tests (MIP, MEP, and HGS) between groups, with lower muscle strength in the sarcopenic group.

**Table 1:** Comparison of anthropometrics, body composition characteristics, and physical tests between groups.

Variable	Non-sarcopenic (n = 66)	Sarcopenic (n = 88)	p value
Age (years)	70.5 (67.0–74.3)	76.0 (69.0–81.0)	0.001*
Height (m)	1.6 (1.5–1.6)	1.5 (1.5–1.6)	< 0.001*
Weight (kg)	69.4 (9.1)	55.0 (7.9)	< 0.001*
BMI (kg/m^2^)	30.0 (4.2)	25.0 (3.8)	< 0.001*
Lean mass (kg)	38.3 (3.1)	31.6 (3.4)	< 0.001*
Fat mass (kg)	28.7 (6.3)	21.0 (5.6)	< 0.001*
BMD (g/cm^2^)	1.0 (0.1)	0.9 (0.1)	< 0.001*
Muscular strength			
MIP (cmH_2_O)	75.0 (55.0–100.0)	60.0 (40.0–83.8)	0.002*
MEP (cmH_2_O)	80.0 (58.8–100.0)	55.0 (35.0–73.6)	< 0.001*
HGS (kg)	23.4 (5.5)	18.0 (4.6)	< 0.001*

Data presented as mean ± SD or median (interquartile range). *BMI* body mass index, *BMD* bone mineral density, *MIP* maximal inspiratory pressure, *MEP* maximal expiratory pressure, *HGS* hand grip strength.*p < 0.05.

### Logistic regression analysis between MIP, MEP, HGS, and sarcopenia

While all the variables being significantly associated with sarcopenia in the univariate analysis [age (OR 1.09; 95% CI 1.04–1.15, p = 0.001), MIP (OR 0.99; 95% CI 0.98–1.00, p = 0.004), MEP (OR 0.97; 95% CI 0.96–0.99, p < 0.001), and HGS (OR 0.79; 95% CI 0.72–0.86, < 0.001)], in the multiple logistic regression with all the variables, only only MEP [OR 0.98; 95% CI 0.97–1.00, p = 0.047] and HGS [OR 0.82; 95% CI 0.75–0.90, p < 0.001] were significantly and inversely associated with sarcopenia (protective impact) (Table 2). Of note, when the data were adjusted for age, the results were the same [OR  1.02; 95% CI 0.96–1.08; p = 0.515].

**Table 2:** Univariate and multivariate logistic regression analysis of sarcopenia.

Variables	Univariate		Multivariate	
	OR (95% CI)	p value	OR (95% CI)	p value
Age (years)	1.09 (1.04–1.15)	0.001*	1.02 (0.96–1.08)	0.538
MIP (cmH_2_O)	0.99 (0.98–1.00)	0.004*	1.00 (0.99–1.01)	0.877
MEP (cmH_2_O)	0.97 (0.96–0.99)	< 0.001*	0.98 (0.97–1.00)	0.047*
HGS (kg)	0.79 (0.72–0.86)	< 0.001*	0.82 (0.75–0.90)	< 0.001*

*OR* odds ratio, *95% CI* 95% confidence interval, *MIP* maximal inspiratory pressure, *MEP* maximal expiratory pressure, *HGS* handgrip strength. *p < 0.05.

Univariate: univariate logistic regression analyses for the association between age, MIP, MEP, HGS, and sarcopenia.

Multivariate: multivariate logistic regression analyses to test whether the MIP, MEP, and HGS are independently associated with sarcopenia.

### ROC curves for MIP, MEP and HGS

**Figure 2:**
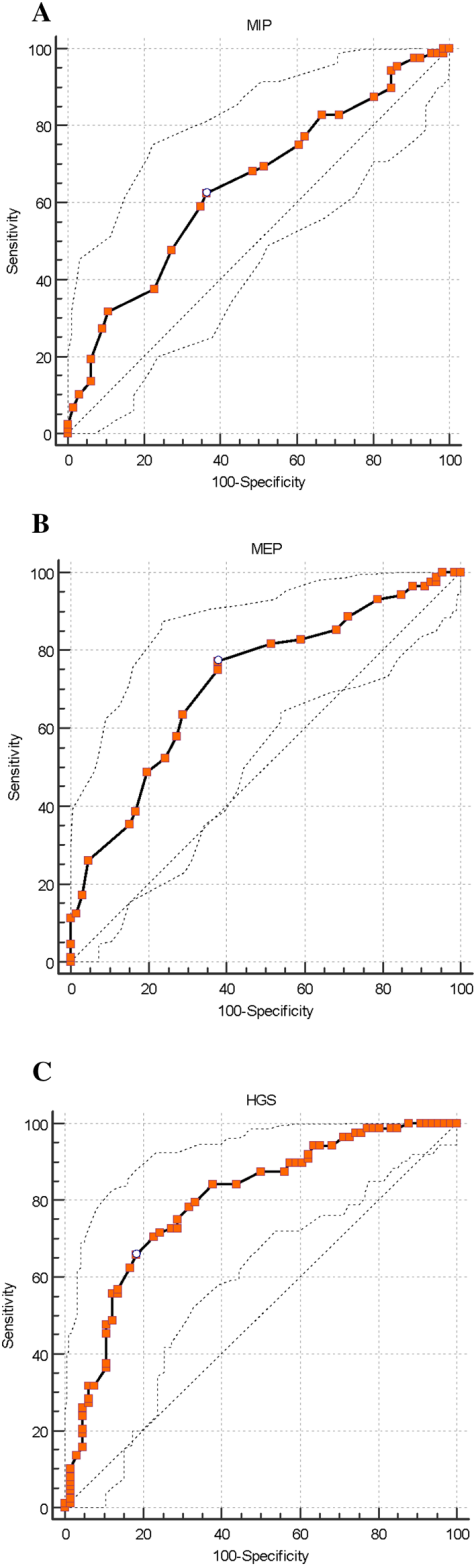
ROC curves for MIP, MEP and HGS. ROC curves for (**A**) MIP [AUC = 0.65 (0.56–0.73)], (**B**) MEP [AUC = 0.72 (0.64–0.80)], and (**C**) HGS [AUC = 0.80 (0.72–0.87)] in the screening of sarcopenia.

The area under the ROC curve (AUC) to exclude sarcopenia in elderly women using strength tests were MIP [AUC = 0.65 (0.56–0.73)], B). MEP [AUC = 0.72 (0.64–0.80)], and C). HGS [AUC = 0.80 (0.72–0.87)]. The MIP showed low discriminatory power while MEP had acceptable accuracy and HGS was excellent for sarcopenia screening (Fig. 2). Table 3 shows properties of the cut-off points with the best combination of sensitivity and specificity, as well negative and positive predictive value of muscle strength tests to screening sarcopenia.

**Table 3:** Cut-off points, AUC, sensitivity, specificity, NPV and PPV of muscle strength tests to screening sarcopenia.

	AUC (95% CI)	Cut-off point	Sensitivity (95% CI)	Specificity (95% CI)	NPV (95% CI)	PPV (95% CI)
MIP	0.65 (0.56–0.73)	67.5	62.5% (51.5–72.6%)	63.6% (50.9–75.1%)	63.6% (54.2–72.0%)	62.5% (55.4–69.1%)
MEP	0.72 (0.64–0.80)	77.0	77.3% (67.1–85.5%)	62.1% (49.3–73.8%)	62.1% (52.9–70.9%)	77.2% (69.9–83.2%)
HGS	0.80 (0.72–0.87)	20.0	70.5% (59.8–79.7%)	77.3% (65.3–86.7%)	77.2% (67.7–84.6%)	70.4% (63.1–76.8%)

*MIP* maximal inspiratory pressure, *MEP* maximal expiratory pressure, *HGS* hand grip strength, *AUC* area under the ROC curve, *NPV* negative predictive value, *PPV* positive predictive value.

## Discussion

To the best of our knowledge, the present study was the first to demonstrate the accuracy of HGS and MEP in identifying older, community-dwelling, Brazilian women with sarcopenia. The main data revealed that sarcopenic, older, community-dwelling women are more likely to have (1) lower respiratory muscle strength, that is, lower MEP and MIP and reduced HGS compared with older community-dwelling women with low muscle mass or sarcopenia. In addition, (2) muscle strength test cut-off points for HGS and MEP appear to be complementary tools for screening sarcopenia in this population. These results are clinically relevant, as simple, low-cost tests have potential value in screening and identifying older, community-dwelling women with sarcopenia, especially when DXA is not available.

The general characteristics of the participants showed differences between the groups regarding anthropometric variables and body composition due to sarcopenia. These findings are consistent with previous studies on aging sarcopenia^36,37^. Regarding muscle strength, both handgrip strength and respiratory muscle strength were lower in individuals with sarcopenia when compared to the non-sarcopenic elderly. Thus, the data of the present study is in accordance with the findings of previous studies that identified lower MIP, MEP, and HGS in older adults with sarcopenia^1,8,22,24,38^.

According to the EWGSOP2, the determination of low muscle strength is essential and the most important component in the diagnosis of sarcopenia^1^. One of the muscle strength tests recommended by the EWGSOP2 with normative reference for healthy, British women is HGS. Thus, the cut-off for healthy British women adults is > 16 kg, based on -2.5 standard deviations from the normative reference mean, that is, healthy British women^1,8^.

In this study, the cut-off point for sarcopenia in elderly, community-dwelling, Brazilian women was 20 kg. A systematic review with HGS reference values in different countries^8^ found that the magnitude of HGS if often related to the level of development of the country. This suggests that the cut-off points from British normative data may not be specific enough in developing regions like Brazil. Nonetheless, the EWGSOP2 recommend the use of regional normative populations when available, given that cut-off values for parameters related to sarcopenia may differ between populations due to ethnicity, body size, lifestyles, and cultural origins^1^.

A few Brazilian studies with different aims have proposed cut-off points for HGS. Sampaio et al.^39^ investigated HGS cut-off values in relation to fear of falling among older, Brazilian adults and the cut-off for women (69.4 ± 6.6 years) was 21.7 kg (AUC = 0.56; 95% CI 0.51–0.62; p = 0.02). De Souza et al.^40^ also showed the cut-off point of HGS to identify mobility limitation in older, community-dwelling people (73.4 ± 6,4 years) and found values of ≤ 17.4 kg (AUC = 0.68; 95% CI 0–64-0.71). These findings are similar to those of the present study since they found higher HGS cut-off points for Brazilian women, but they differ in terms of the specific values and outcomes of interest assessed. Although such clinical outcomes are related to sarcopenia, it should be noted that the AUC had low discriminatory power in both studies (AUC < 0.7).

Of the analyzed tests, only MEP and HGS presented a satisfactory area under curve that enabled the ROC curve to be calculated. The cut-off point for MEP was 77 cmH_2_O (sensitivity: 77.3%; specificity: 62.1%; AUC: 0.72) and the HGS was 20 kg (sensitivity: 70.5%; specificity: 77.3%; AUC: 0.80). As in the EWGSOP2, we opted to use rounded figures because, despite minimally reducing the accuracy, it makes their use easier. Therefore, we propose the values of ≤ 77 cmH_2_O for MEP and ≤ 20 kg for HGS for identifying sarcopenia in older, community-dwelling, Brazilian women.

Some studies have already investigated the relationship between respiratory muscle strength and sarcopenia. Shin et al.^23^ verified the relationship between MIP and MEP and sarcopenia indicators (skeletal muscle mass index, HGS, gait speed, and SPPB) among 65 elderly people with a mean age of 69.90 ± 7.63 years. Both MIP and MEP were positively related to skeletal muscle mass index and HGS. Izawa et al.^22^ also proposed a cut-off point for MIP as a discriminator of sarcopenia, with a value of 55.6 cmH_2_O (sensitivity: 0.76; specificity: 0.37; AUC: 0.70). However, the individuals included in the analysis were elderly male patients with heart disease, so sex-related differences could not be evaluated. This limits the use of this cut-off point with females and individuals without heart disease^41,42^.

Ohara et al.^38^ also developed cut-off values of MIP and MEP for the diagnosis of sarcopenia in elderly Brazilians^22,38^. In this study, the cut-off points ≤ 45 cmH_2_O for MIP (sensitivity: 0.73; specificity: 0.64; AUC: 0.73) and ≤ 55 cmH_2_O for MEP (sensitivity: 0.73; specificity: 0.58; AUC: 0.71) were discriminators of sarcopenia in elderly women. However, the authors evaluated the muscle mass component based on the total muscle mass estimated by the equation proposed by Lee et al.^43^. In this regard, although the equation proposed by Lee is validated for the European population, it is not a gold standard to measure muscle mass, especially for the Brazilian population.

Another recent study demonstrated that frail and pre-frail older adults present significantly lower MIP and MEP compared to non-frail older people^44^, indicating that respiratory muscle strength may be useful for discriminating frailty^34^. This reinforces the importance of considering respiratory muscle strength to assess frailty in older adult populations. Together, these results demonstrate the importance of the relationship between respiratory muscle strength and sarcopenia indicators.

Although the present study evaluated MIP and MEP, only MEP was considered an acceptable clinical test for identifying sarcopenia. A possible explanation could be that expiratory muscles seem to be more vulnerable to the aging process than inspiratory muscles^45–47^. Black and Hyatt^48^ observed that respiratory muscle strength declines at a rate between 0.25 and 0.79 cmH_2_O a year for MIP and between 1.14 and 2.33 cmH_2_O a year for MEP in both men and women, respectively. Enright et al.^21^ also found similar age-related decrements in both MIP and MEP with a rate of decline in MIP of about 1 cmH_2_O a year and for MEP of about 2 to 3 cmH_2_O a year for individuals between 65 and 85 years of age.

Our findings suggest that respiratory muscle strength is a relevant component in the clinical evaluation of the elderly due to its association with sarcopenia. In addition, MEP and HGS are easily accessible measures performed in clinical practice and can be useful to provide information about the health status of the elderly population. Advances in research are needed for external validation of the cut-off points proposed by the present study.

This study has several strengths that are worth highlighting. First, we used DXA, a measure considered the gold standard for measuring muscle mass and which is recommended in research and clinical practice by the EWGSOP2 to confirm a sarcopenia diagnosis^1^. Furthermore, this was the first study to establish cut-off points for HGS and one of the first for MEP in identifying sarcopenia in older, community-dwelling, Brazilian women. It is also noteworthy that the fact elderly women from different locations were recruited enabled us to obtain data that more reliably portray the diversity of the residents of this community.

The Southeast Region of Brazil is a region with multiple local realities, including physical, social, and economic characteristics, therefore the results of the present study should be read with caution and cannot be extrapolated to all Brazilian elderly women. In addition, results cannot be extrapolated to elderly women who engage in physical activity on a regular basis because we included only sedentary elderly in our study. Limitations of this cross-sectional study include the inclusion of female subjects only, so gender-related differences could not be assessed. Future studies evaluating factors such as MEP and HGS in longitudinal settings, performed for longer periods, will be required to determine the risk of developing sarcopenia.

## Conclusion

MEP and HGS are probably valuable tools with potential value in the screening of sarcopenia in older, community-dwelling, Brazilian women. Cut-off points of ≤ 77 cmH_2_O for MEP and ≤ 20 kg for HGS seem to be useful and should be considered for the identification of sarcopenia in the studied population. Future work is still needed to assess external validation of the proposed cut-offs before the clinical application.

## Data Availability

The datasets generated and/or analyzed during the current study are not publicly available due the maintenance of confidentiality of our participants and declarations within the written information which participants had agreed on, but are available from the corresponding author on reasonable request.
